# On the Vesicating Properties of the Weevil

**Published:** 1839-11-23

**Authors:** 


### *On the Vesicating Properties of the Weevil. Extracted from the Inaugural Essay of* WM. M. S. RIDLEY, M. D., of North Carolina.

I propose offering to the consideration of the Medical Faculty of the University of Pennsylvania, the results of some experiments made by me for the purpose of ascertaining the vesicating powers of an insect, commonly known by the name of *Weevil*, and in Natural History by that of *Calandra granaria.*

A scientific description of this small insect may be found in the 12th volume of the Encyclopœdia Americana, under the head of Curculis. It is best known in the south from the great havoc it produces among crops of wheat and other small grain. Its ravages are not confined to the grain while maturing, but even after it is harvested the farmer encounters the greatest difficulty in expelling this intruder from his granaries.

"The *Calandra granaria* is a native of Europe, whence it has probably been introduced into this country in the grain which has been from time to time imported. Immense quantities of the insect are now found in the southern parts of the United States; and its devastating influence is felt in all those sections in which grain is the chief article of cultivation. It may generally be found, in swarms, in beds of wheat, corn, rye, or oats, and more especially when the grain is garnered in warm barns, which have been in use for some length of time. It is not to be seen in the open air in autumn or winter, as it goes into winter quarters early in the fall, and is not out again until late in the ensuing spring.

"In the fall of 1836, in the State of North Carolina, the author was overlooking some negroes, who were stirring and removing a quantity of wheat from one granary to another. This occasioned much disturbance among the insects. The weevil, in seeking a place of refuge, would alight, indiscriminately, in every direction, and particularly upon the bare necks and hands of the persons engaged in removing their beds. The negroes would either brush them away or crush them upon the surface. I noticed, on the subsequent day, that all those who had been thus engaged, were much spotted and blistered upon the parts of their bodies which had been exposed. This induced me to inquire of them the cause of the peculiar appearance, when one of the most intelligent of them informed me that it was a necessary consequence of thrashing, or otherwise interfering with wheat after it had been garnered. Being rather incredulous, I made some inquiries of intelligent farmers, who informed me that it was a fact which they had long noticed, but could not account for.

"Having determined to investigate the affair, I made some experiments with the insects, and found that, upon being crushed upon the naked skin, they had the effect of producing vesications to a considerable degree. I also found that the effect was greatest immediately at the spot where the insect was crushed, and that the more equally the resulting fluid was diffused over the surface, the less was the injury done to the skin. These facts induced me to try the efficacy of the weevil as a vesicating agent.

"Having collected some of the insects, and exposed them to the sun's rays, for the purpose of drying them, I prepared them into a cerate, according to the directions of the United States Pharmacopœia for "Ceratum Cantharides." A portion of this preparation I presented to my preceptor, Dr. James Ridley, with the request that he would employ it on the first opportunity, and inform me of the result. In a few days an opportunity was offered, and the cerate made from the weevils was substituted for the ordinary blistering cerate made with Spanish flies. It was found to produce identically the same effect as the latter, causing in a short time redness of the part, and in the course of a few hours, full vesication, without any symptoms of strangury. Since the time above adverted to, the author has had it in his power to employ the remedy only once; and, in this instance, it was desirable to obtain only its rubefacient action. The application was made over the epigastrium, and the effect desired was produced in a few hours. From these facts it may be inferred that the therapeutical operation of the weevil, as an external remedy, is the same as that of the Spanish or potato fly. Its internal use may also be found identical, but the author has yet made no trials with it in this way. He only wishes to call attention to the subject, as it certainly presents an ample field for investigation, and may lead to curious if not useful results.

"The only difficulty attending the investigation is that of taking the insects without injuring them, as upon this depends their efficiency as a remedy. The best method is to build up a large fire in the barn in which they may reside, when, if aroused from their beds in the grain and crevices of the granary, they will assemble in swarms towards the centre of the building, immediately over the fire, and will then fall down, either from the effects of the heat or from suffocation. After this, they may be collected and exposed for drying to the sun's rays. For use, they may be powdered, and treated precisely as the Spanish fly, in the preparation of the blistering cerate."

*Extract from a Letter of Dr. Ridley, to Prof. Wood, of this City, dated Columbus, Georgia, November 3d*, 1839.

"Since obtaining my degree, I have continued to use the cerate made from the *Calandra granaria*, and it gives me much pleasure to assure you that my expectations have been even surpassed. I am so fully convinced of its superiority as a vesicative agent over that prepared from the Spanish or potato fly, that I have adopted it in my practice, to the almost entire exclusion of the cerate in common use. My opinion is supported by numerous practitioners who have noticed its effects, and pronounce their preference for it over any other vesicating ointment they have ever used. It will produce a blister sooner than any other ointment, without those distressing symptoms so commonly attendant upon the action of cantharides."

